# Supplementary material for: Transcriptional profiling predicts running promotes cerebrovascular remodeling in young but not midlife mice
Source: BMC Genomics. 2019 Nov 14;20:860. doi: 10.1186/s12864-019-6230-z (PMC6854620; doi:10.1186/s12864-019-6230-z)
Supplement: Supplementary file 1 — Additional file 1: Figure S1. Midlife runners show variation in running speed and distance. Figure S2. Midlife High runners show reduced adiposity due to running. Figure S3. CPM scatterplot of young runners compared to young sedentary in the cortex and hippocampus. Figure S4. CPM scatterplot of midlife runners compared to midlife sedentary in the cortex and hippocampus. Figure S5. Fewer DE genes in the cortex and hippocampus of midlife run mice. Figure S6. Col4a1, Lamb2 and Lama5 are expressed in multiple cerebrovascular-related cells. [file 12864_2019_6230_MOESM1_ESM.docx]

**Figure S1: Midlife runners show variation in running speed and distance.** (A) Significant difference in distance ran between midlife low running and midlife high running groups (p>0.001). (B) Low runners spent less time (% of time) running than high runners. (C) Midlife High runners ran faster, with a higher percentage of time running above 100 rotations per minute. (D) Final weight at week 12 showed a significant difference in young sedentary and young runners (**p =0.0018).

**Figure S2: Midlife High runners show reduced adiposity due to running.** (A) Weight of midlife mice at time of NMR showed no significant difference. (B) Significant reduction in amount of fat (grams) in midlife high compared to midlife sedentary mice (*p =0.0225). (C) No significant difference in lean mass (grams) between the midlife cohorts. (D) Significant reduction in adiposity (%fat) in midlife high runners compared to midlife sedentary (**p= 0.0060). (E) Significant increase in lean muscle percentage of midlife high runners compared to midlife sedentary (*p= 0.0154).

**Figure S3: CPM scatterplot of young runners compared to young sedentary in the cortex and hippocampus.** (A-B) Counts per million (CPM) scatterplot of young runners compared to young sedentary in the cortex (A) and hippocampus (B) revealed Mouse 18606 as an outlier from the hippocampal dataset. This sample was removed from further analysis.

**Figure S4: CPM scatterplot of midlife runners compared to midlife sedentary in the cortex and hippocampus.** (A-B) Counts per million (CPM) scatterplot of midlife runners compared to midlife sedentary in the cortex (A) and hippocampus (B) revealed no outliers in this dataset. There was also no observable difference between high runners and low runners.

**Figure S5: Fewer DE genes in the cortex and hippocampus of midlife run mice.** (A) Number of DE genes (FDR <0.05) in the cortex of young running compared to young sedentary, and all midlife running mice compared to midlife sedentary. (B) Number of DE genes (FDR <0.05) in the hippocampus of young running compared to young sedentary, and all midlife running mice compared to midlife sedentary.

**Figure S6: *Col4a1*, *Lamb2* and *Lama5* are expressed in multiple cerebrovascular-related cells.** (A) Cell type specific expression from Zheng *et. al*. (left), and Vanlandewijck and He *et. al*. and He *et. al.* (right), showing overall expression of *Col4a1* in endothelial cells, smooth muscle cells, pericytes and fibroblast-like cells. (B) Expression of *Lamb2* in endothelial cells, smooth muscle cells, pericytes and astrocytes. (C) Expression of *Lama5* in endothelial cells and astrocytes.
